# Artificial Intelligence in Orthopaedic Training: A Narrative Literature Review of Applications, Evidence, and Limitations

**DOI:** 10.7759/cureus.113975

**Published:** 2026-08-04

**Authors:** Bethany Sykes, Adan Khan

**Affiliations:** 1 Trauma and Orthopaedics, Salisbury NHS Foundation Trust, Salisbury, GBR

**Keywords:** artificial intelligence (ai), orthopaedics and trauma, simulation in medical education, surgical-education, ­trauma

## Abstract

Orthopaedic surgical training has traditionally relied on an apprenticeship model, but reduced working hours and heightened patient safety expectations have narrowed the operative exposure available to trainees, prompting growing interest in adjuncts to conventional training. Artificial intelligence (AI) is increasingly being applied within surgical education as one method of supporting learning, offering new approaches to simulation, technical skills assessment, and personalised learning. Given the rapid pace of development in this field, this narrative review synthesises the current evidence for AI applications in Trauma & Orthopaedics training, examining three key domains: AI-enhanced simulation, objective skills assessment, and predictive analytics for personalised learning pathways.

The evidence indicates consistent, if preliminary, benefit from AI-enhanced virtual reality (VR) simulation, including improved arthroscopy skills and reduced operative duration, alongside promising applications of haptic feedback for real-time coaching and personalised, algorithmically generated performance feedback. Objective, motion-tracking-based skills assessment significantly improved with established tools such as Objective Structured Assessment of Technical Skills (OSATS) and Global Evaluative Assessment of Robotic Skills (GEARS), though this evidence largely originates from general and robotic surgery rather than orthopaedics specifically. Predictive analytics offer early potential to forecast trainee learning curves and identify those at risk of underperformance but raise ethical concerns regarding premature labelling and its effect on trainee confidence and progression.

Across all domains, evidence remains limited by small, single-institution studies, a lack of randomised controlled trials, and a near-total absence of research linking AI-assessed simulation performance to long-term patient outcomes. Cost and unequal access to high-fidelity simulation technology have the potential to further constrain widespread adoption. AI therefore holds considerable promise for orthopaedic training but should currently be regarded as a complement to, rather than a replacement for, traditional apprenticeship-based education, pending stronger longitudinal and comparative evidence.

## Introduction and background

Artificial intelligence (AI) is a widely used term referring to technology capable of simulating human learning, comprehension, problem-solving, decision-making, creativity, and autonomy [1]. AI systems can process huge volumes of data, identifying patterns and completing complex analyses that would be impractical for a human to perform manually. The field can be broadly subdivided into three approaches: symbolic AI, which uses logic and knowledge representation; statistical AI, which applies probability and machine learning (ML); and sub-symbolic AI, which uses search-based and neural-network methods. It encompasses ML, deep learning (DL), and neural networks and has become of significant interest as a tool for developing medical education, particularly within surgical specialties [1,2].

Surgical training has traditionally followed an apprenticeship model, in which trainees acquire technical competence through graduated, supervised exposure to real patients under the guidance of a senior surgeon [3]. While this model has underpinned surgical education for well over a century, it has come under increasing strain in recent decades. Reforms to working patterns, including statutory limits on trainee working hours in areas, have substantially reduced the cumulative operative exposure available to a given trainee during their training period [4]. These pressures have created a widening gap between the volume of hands-on experience trainees are able to accrue and the level of competence expected of them by the end of training, prompting sustained interest in adjuncts and alternatives to traditional operative exposure [4].

Orthopaedic surgery is a specialty in which this tension is felt particularly acutely. It demands a wide range of skills and knowledge, encompassing complex three-dimensional anatomy, high procedural precision, frequent use of specialised instrumentation, and considerable cognitive load, often under time pressure in trauma settings [2]. Many core orthopaedic procedures, such as arthroscopy, have a recognised and often steep learning curve, meaning early attempts by trainees carry a genuine risk of prolonged operative time or suboptimal outcomes if undertaken without adequate preparation [5,6]. This has historically limited the extent to which trainees can practise such procedures on real patients before achieving a baseline level of competence, creating demand for training methods that allow rehearsal without direct patient risk [5,6].

Simulation-based training, in various forms, has emerged as a partial response to this need, allowing trainees to rehearse technical skills in a controlled, repeatable environment. However, conventional simulators, whether physical models or early virtual reality (VR) platforms, have generally offered a fixed, non-adaptive training experience, covering the same scenarios and difficulty level regardless of the individual trainee's baseline ability or rate of progress. This limits their capacity to provide truly individualised training and means that exposure to rarer or more complex case presentations remains a matter of chance rather than deliberate curriculum design [2].

It is against this backdrop that AI-based tools for orthopaedic education have developed rapidly, including VR surgical simulators with adaptive learning modules that allow trainees to practise challenging or complex procedures without risk to patient outcomes [2]. By enabling systems to adjust scenario difficulty, generate varied case presentations, and provide automated, individualised feedback, AI offers a potential means of addressing several of the limitations inherent in both traditional apprenticeship training and earlier-generation simulation [5-7]. Beyond technical skill acquisition, AI-driven platforms are also being applied to knowledge-based learning and to the analysis of trainee performance data more broadly, raising the possibility of more personalised, data-driven training pathways across orthopaedic training as a whole [5,6].

Given the pace at which this field is developing, the growing volume of relevant literature, and the significant potential for improving medical education, this narrative review sets out to synthesise the current evidence for AI applications in orthopaedic surgical training. It examines simulation-based training, objective skills assessment, and predictive analytics for personalised learning before critically appraising the strengths and limitations of the existing evidence base. In particular, it addresses a notable gap in the literature: despite growing interest in AI-enhanced training, there is a lack of evidence linking AI-assessed simulation performance to long-term, real-world patient outcomes.

## Review

Methodology

A literature search was conducted to identify studies examining the application of AI in orthopaedic training and education. Electronic databases, including PubMed, Embase, and Web of Science, were searched for relevant articles published between 2000 and 2026. The search strategy combined Medical Subject Headings (MeSH) terms and free-text keywords related to three core concepts: AI (e.g., "artificial intelligence," "machine learning," "deep learning," "neural network," "virtual reality," "augmented reality," "haptic feedback"), orthopaedics (e.g., "orthopaedic," "orthopedic," "musculoskeletal," "trauma surgery"), and training or education (e.g., "surgical training," "simulation," "skill acquisition," "competency assessment"). Boolean operators (AND/OR) were used to combine search terms, and reference lists of included articles were hand-searched to identify additional relevant studies.

Studies were included if they evaluated an AI-based tool, platform, or system used for orthopaedic surgical training or education, were published in a peer-reviewed journal or conference proceeding, were available in English, and reported original data or a review relevant to the research question. Studies were excluded if they focused solely on AI applications in clinical diagnosis or treatment without a training/educational component, were conference abstracts, editorials, or non-peer-reviewed commentaries lacking sufficient methodological detail, or did not specifically address orthopaedic training.

Given the anticipated heterogeneity in study designs, AI applications, and outcome measures, findings were synthesised narratively rather than through systematic review and meta-analysis. Studies were grouped thematically according to the type of AI application (e.g., simulation-based training, haptic feedback systems, VR platforms, ML-based performance assessment) to facilitate comparison across the literature and identify trends, gaps, and future research directions.

AI-enhanced simulation and VR training

Research has demonstrated that VR and AI-enhanced simulation can improve technical skills. The use of VR by orthopaedic trainees has been shown to benefit their outcomes both objectively and subjectively [8-11]. A study found that VR use improved arthroscopy skills, acknowledged to be a particularly difficult skill for trainees to gain experience in, and decreased operative duration, while trainees themselves reported higher confidence in transferring these simulated skills to operative settings [9]. This offers a safe and widely applicable educational tool to complement traditional training.

A defining feature distinguishing AI-enhanced simulation from earlier generations of VR trainers is adaptivity. Rather than presenting a fixed set of scenarios, modern platforms can generate a wide variety of training cases, from common procedures to rare or complex presentations, and adjust the difficulty of subsequent tasks according to a trainee's ongoing performance [12-14]. This allows challenge to be calibrated to the individual, encouraging accelerated learning curves while also providing exposure to rare presentations that trainees would otherwise seldom encounter in clinical practice [15]. In principle, this individualised difficulty-scaling addresses a long-standing limitation of traditional training, in which exposure to complex or unusual cases depends largely on chance and caseload rather than deliberate, structured practice [14].

A further development has been the integration of haptic feedback systems with ML to support real-time coaching during simulated procedures. A haptic feedback system is a technology that uses motors, actuators, or resistive mechanisms to simulate the sense of touch, such as force, vibration, or resistance, allowing users to physically feel simulated interactions like bone density or tissue resistance during virtual or robotic-assisted procedures. Several orthopaedic-specific simulators now combine force-feedback hardware with algorithms that detect deviations from ideal technique as they occur, rather than relying solely on post surgery review [14,16]. Examples include haptic-enabled simulators developed specifically for femoral nailing and orthopaedic drilling, in which ML models classify a trainee's movement style in real time and generate corrective haptic cues to guide instrument trajectory, force application, or drilling depth back toward an expert-defined optimum [16,17]. Similar approaches have been applied to bone-sawing tasks, where force-feedback simulators have been used to distinguish novice from experienced performance and provide tactile guidance during the procedure itself [10,18]. These systems represent a shift from feedback as a discrete, end-of-session event delivered by a supervisor, toward continuous, algorithmically mediated coaching embedded within the simulation itself [14,16-18].

However, evidence for the transfer of these simulated gains into genuine operating theatre performance remains the weakest link in this literature, and this warrants particular caution. Much of the supporting evidence for VR and haptic-enhanced training relies on improvements measured within the simulator itself, or on trainees' self-reported confidence, rather than on objectively measured performance during real procedures [19]. Where transfer has been directly compared - for instance between haptic-enabled VR simulators and simpler box-trainer alternatives in laparoscopic training - no significant difference in skill transfer to the operative environment has been found between approaches, despite VR's greater technological sophistication and cost [10]. This suggests that increased simulator fidelity does not automatically translate into superior real-world skill transfer, and that the causal chain from simulated practice to safer, more competent live surgery remains poorly evidenced, particularly within orthopaedics specifically [10].

A key limitation in the wider application of AI-enhanced simulation, beyond questions of efficacy, is high cost, which is considered further below alongside broader equity concerns.

Objective skills assessment

AI is rapidly developing as a method of objective surgical performance measurement, moving away from subjective, trainer-dependent scoring toward automated, data-driven measures of technical performance. Computer vision and motion-tracking systems have been used to capture instrument trajectories, hand movements, and economy of motion during simulated and real orthopaedic procedures, generating quantitative metrics such as path length, smoothness, and time-to-completion [20-22]. In robotic surgery specifically, DL-based instrument tracking has been used to derive motion metrics directly from operative video, with skill-prediction models built on these metrics able to classify trainees against traditional grading thresholds [22]. Although much of this evidence originates from general, urological, and robotic-assisted procedures rather than orthopaedics directly, the underlying techniques are increasingly being proposed as transferable to orthopaedic contexts such as arthroscopy, where similar demands on instrument handling and bimanual dexterity apply [23].

These automated approaches are typically benchmarked against established rating tools, particularly the Objective Structured Assessment of Technical Skills (OSATS) and the Global Evaluative Assessment of Robotic Skills (GEARS) [23]. Both were originally designed to reduce subjectivity by scoring trainees against structured criteria such as respect for tissue, time and motion, and instrument handling, but they remain dependent on expert observation and are time-consuming to administer at scale [24]. Studies comparing AI-derived scores against these tools have reported encouraging results, with automated skill-prediction models achieving classification accuracies in the region of 80% or higher against OSATS and GEARS thresholds [24,25]. This suggests AI-based scoring may address one of the longstanding weaknesses of traditional assessment: inconsistency between human examiners.

However, a critical appraisal of this literature identifies important limitations in generalisability [26]. Reported accuracies vary considerably depending on which skill domain is assessed, ranging from around a third to over 90% depending on the metric and dataset used [24]. This indicates that performance is highly sensitive to the specific task, sensor setup, and patient population studied. Most validation work has taken place in single-institution studies using specific robotic platforms, most notably the da Vinci system, and it remains unclear whether models trained on one procedure, surgeon population, or device generalise to the more varied instrumentation and open or arthroscopic techniques common in orthopaedic training. This raises a central tension in the field: AI-based objective assessment shows strong internal validity within the narrow contexts in which it has been tested, but external validity across surgeons, procedures, and institutions remains largely unproven, particularly for orthopaedic-specific applications where dedicated evidence is still sparse [27].

Predictive analytics and personalised learning pathways

Across medical education, AI-driven learning platforms are increasingly being used to deliver individualised and adaptive education [28]. These tools can be used to develop targeted case scenarios, deliver questions, and offer personalised explanations as required, tracking and analysing past performance, targeting weaknesses, and adjusting question difficulty to enhance attainment [28].

Beyond question-based learning, a growing body of work has applied ML directly to technical skill acquisition, aiming to predict a trainee's learning curve from only their earliest attempts at a task [29]. Traditional models of proficiency-gain, such as log-linear curves or cumulative summation (CUSUM) analysis, describe a trainee's trajectory only in retrospect and cannot forecast how long an individual will take to reach competence, since proficiency gain is rarely linear and varies considerably between trainees according to their underlying aptitude [30]. Newer supervised and ensemble ML models attempt to address this by extracting a small number of features from initial practice trials to characterise a trainee's overall "learning ability", with some models able to predict learning curve characteristics robustly from only the first few attempts and even to generalise these predictions across different simulation platforms [31]. Similar approaches using AI-selected performance metrics have been used to distinguish learning curves between novices, residents, and experts on simulated tasks, suggesting these models can meaningfully stratify trainees by developmental stage rather than treating skill acquisition as a uniform process [32].

A related and more ethically sensitive application is the early identification of trainees at risk of failing to reach independent competence. Survey-based estimates suggest a meaningful minority of surgical residents struggle to reach the expected level of technical competence within standard training timeframes, and predictive models raise the possibility of flagging such trainees earlier than traditional apprenticeship-based observation would allow, enabling additional targeted support [33]. A commonly cited concern is the risk that this predictive capability, while intended to support struggling trainees, could instead function as a form of premature labelling [33]. Being algorithmically identified as "at risk" early in training carries the potential to affect a trainee's confidence, their relationship with supervisors, and future career progression, particularly if predictions are made from limited early data or fail to account for individual variation in learning trajectories [32]. Predictive AI models could, in theory, also be used to inform decisions around readiness for independent, unsupervised practice, but translation of this into real-world decision-making has not yet been adequately tested.

Challenges in AI integration and limitations

A recurring theme across multiple studies was the need for a cautious and thoughtful approach in managing the ethical issues and challenges arising from the rollout of AI systems. In some areas, the literature strongly supports the benefits of AI application, including skills assessment [34,35]. However, there is less evidence to support the long-term benefit of this in practice, which may indicate both a publication bias toward positive results and genuine challenges in objectively assessing benefit in non-simulated healthcare settings.

Many studies are limited by small sample sizes and single-institution designs and, due to the nature of the research, cannot be undertaken using more robust methodologies such as randomised controlled trials.

A significant and underexplored challenge concerns data privacy and consent. Many AI-driven assessment tools rely on recording and analysing trainee performance, whether through operating-room video, motion-tracking sensors, or simulation logs, and this raises distinct ethical questions beyond those typically considered in patient data governance [34,35]. Consent processes for such recordings frequently fail to specify important details, including who will have access to the footage, how long it will be retained, and for what purposes it may later be used, leaving considerable ambiguity for both trainees and patients [34]. Ownership of recorded material is similarly contested: some have argued surgeons hold a legitimate claim to footage documenting their own operative skill, while others propose shared ownership between surgeons, institutions, and patients, particularly where footage could later be monetised or repurposed for AI model training [35]. Beyond consent and ownership, there are practical confidentiality risks, since recordings may inadvertently capture identifiable patient information or incidental discussion of other patients' care during lengthy procedures [34,35]. For trainees specifically, an additional concern is the potential for recorded performance data to be used punitively in credentialing or progression decisions beyond its original educational purpose, a concern compounded by the current lack of standardised institutional policy on how such footage should be stored, secured, and governed [36]. Emerging approaches such as decentralised or privacy-preserving analysis of surgical video have been proposed as a partial technical solution, but these remain at an early stage of development and are not yet embedded in mainstream orthopaedic training programmes [37].

Concerns are also repeatedly raised about equity of access to expensive AI-enhanced training, particularly simulation, globally. Implementing advanced AI systems requires significant resources and infrastructure, and high costs may exacerbate healthcare inequalities due to disparities in availability between well-funded institutions and others [36].

The incorporation of VR is accompanied by its own set of challenges and significant costs. There are many types of VR simulators available on the market, which makes it difficult to conclusively compare and draw conclusions about the success of VR across all brands and types. The inclusion of VR in simulation is also associated with significantly higher costs than lower-fidelity alternatives, and this disparity can usefully be considered across the dimensions of availability, accessibility, and affordability [38]. At present, high-fidelity systems remain concentrated in well-funded academic centres: one survey of orthopaedic surgeons found that only 3% of trainees had had exposure to VR training, and high-end platforms such as haptic-feedback simulators can cost upwards of $100,000-200,000 [39,40]. However, in other medical and surgical fields, this pattern of concentrated early adoption is consistent with the broader trajectory of medical technology diffusion, in which early uptake by high-volume, well-resourced institutions has historically driven the standardisation, validation, and technical refinement that precede wider scalability [41]. Consistent with this trajectory, VR hardware costs have fallen substantially over time as the technology has matured, with off-the-shelf commercial headsets now available for a small fraction of the cost of early systems, enabling low-cost simulation platforms that were not previously feasible [40,42]. Whether this trend will translate into genuine affordability and accessibility for lower-resourced training programmes, rather than a widening gap as academic centres continue to adopt newer and more expensive iterations, remains uncertain, and studies have suggested current systems may nonetheless be cost-effective, though the need for more thorough costing is highlighted [38]. Almost all studies acknowledge that, in its current state, VR is unable to offer a complete training experience and is best used as a supplemental resource to enhance traditional methods.

Additionally, there remains a notable gap in the literature on the effect of AI-enhanced training on long-term patient outcomes in real healthcare settings, as longitudinal studies linking AI-assessed simulation performance to real-world patient outcomes are notably absent.

## Conclusions

AI-driven tools show real but preliminary promise in trauma and orthopaedic training, particularly VR-enhanced simulation and computer-assisted skills assessment, which are linked to better technical performance, shorter simulated operative times, and increased trainee confidence. Personalised, adaptive feedback platforms also show early potential for tailoring both technical and knowledge-based learning. Yet this evidence rests almost entirely on simulated settings, as few studies are adequately powered, multi-centre, or methodologically rigorous, and none yet demonstrate that AI-assessed simulation performance translates into better long-term patient outcomes. Closing this evidence gap through longitudinal, multi-institutional research should be the field's top priority.

Progress is also constrained by unresolved ethical and governance issues. Performance-recording tools raise questions about consent, data access, retention, ownership, and the risk of footage being monetised or repurposed for future model training. There is a further danger that data gathered for educational purposes could be used punitively in trainee progression decisions, especially given the lack of standardised data governance. Combined with cost and access barriers to high-fidelity VR systems, these concerns mean AI should currently be regarded as a complement to, not a substitute for, traditional training. Until more robust longitudinal evidence and clear frameworks for consent, ownership, and data use are established, orthopaedic training programmes should adopt these technologies cautiously and incrementally, balancing their genuine promise against the current limits of the evidence.

## References

[1] Szatkowski JP, Druten E, Soni C, O'Neill DC (2025). Artificial intelligence in orthopaedic education: a narrative review. Ann Jt.

[2] Koucheki R, Lex JR, Brock M, Goel DP (2025). Integrating artificial intelligence and virtual reality in orthopedic surgery: a comprehensive review. HSS J.

[3] Khan MR, Begum S (2021). Apprenticeship to simulation - the metamorphosis of surgical training. J Pak Med Assoc.

[4] Wyman MG, Huynh R, Owers C (2022). The European Working Time Directive: will modern surgical training in the United Kingdom be sufficient?. Cureus.

[5] Leal JA (2025). Artificial intelligence in orthopaedic and trauma surgery education: applications, ethics, and future perspectives. J Am Acad Orthop Surg Glob Res Rev.

[6] Al-Saadawi A, Tehranchi S, Ahmed S, Nzeako OJ (2025). Exploring the current applications of artificial intelligence in orthopaedic surgical training: a systematic scoping review. Cureus.

[7] Banskota B, Bhusal R, Yadav PK, Banskota AK (2025). Artificial intelligence in orthopaedic education, training and research: a systematic review. BMC Med Educ.

[8] Pedowitz RA, Marsh JL (2012). Motor skills training in orthopaedic surgery: a paradigm shift toward a simulation-based educational curriculum. J Am Acad Orthop Surg.

[9] Cannon WD, Nicandri GT, Reinig K, Mevis H, Wittstein J (2014). Evaluation of skill level between trainees and community orthopaedic surgeons using a virtual reality arthroscopic knee simulator. J Bone Joint Surg Am.

[10] LeBlanc J, Hutchison C, Hu Y, Donnon T (2013). A comparison of orthopaedic resident performance on surgical fixation of an ulnar fracture using virtual reality and synthetic models. J Bone Joint Surg Am.

[11] Seymour NE, Gallagher AG, Roman SA, O'Brien MK, Bansal VK, Andersen DK, Satava RM (2002). Virtual reality training improves operating room performance: results of a randomized, double-blinded study. Ann Surg.

[12] Gasco J, Patel A, Ortega-Barnett J (2014). Virtual reality spine surgery simulation: an empirical study of its usefulness. Neurol Res.

[13] Hooper J, Tsiridis E, Feng JE (2019). Virtual reality simulation facilitates resident training in total hip arthroplasty: a randomized controlled trial. J Arthroplasty.

[14] Sugand K, Akhtar K, Khatri C, Cobb J, Gupte C (2015). Training effect of a virtual reality haptics-enabled dynamic hip screw simulator. Acta Orthop.

[15] Lohre R, Bois AJ, Athwal GS, Goel DP (2020). Improved complex skill acquisition by immersive virtual reality training: a randomized controlled trial. J Bone Joint Surg Am.

[16] Tsai MD, Hsieh MS, Tsai CH (2007). Bone drilling haptic interaction for orthopedic surgical simulator. Comput Biol Med.

[17] Syamlan AF, Denis K, Poorten EV (2022). Haptic/virtual reality orthopaedic surgical simulators: a literature review. Virt Real.

[18] Lin Y, Wang X, Wu F, Chen X, Wang C, Shen G (2014). Development and validation of a surgical training simulator with haptic feedback for learning bone-sawing skill. J Biomed Inform.

[19] Hasan LK, Haratian A, Kim M, Bolia IK, Weber AE, Petrigliano FA (2021). Virtual reality in orthopedic surgery training. Adv Med Educ Pract.

[20] Carciumaru TZ, Tang CM, Farsi M (2025). Systematic review of machine learning applications using nonoptical motion tracking in surgery. NPJ Digit Med.

[21] Zia A, Sharma Y, Bettadapura V, Sarin EL, Essa I (2018). Video and accelerometer-based motion analysis for automated surgical skills assessment. Int J Comput Assist Radiol Surg.

[22] Lee D, Yu HW, Kwon H, Kong HJ, Lee KE, Kim HC (2020). Evaluation of surgical skills during robotic surgery by deep learning-based multiple surgical instrument tracking in training and actual operations. J Clin Med.

[23] Castillo-Segura P, Fernández-Panadero C, Alario-Hoyos C, Muñoz-Merino PJ, Delgado Kloos C (2021). Objective and automated assessment of surgical technical skills with IoT systems: a systematic literature review. Artif Intell Med.

[24] Laughlin JL, Johnson LR, Ghanekar B, O'Malley MK (2025). Technical skills assessment in robotic surgery: a review of recent methods. Methodist Debakey Cardiovasc J.

[25] Lam K, Chen J, Wang Z (2022). Machine learning for technical skill assessment in surgery: a systematic review. NPJ Digit Med.

[26] Boal MW, Anastasiou D, Tesfai F (2024). Evaluation of objective tools and artificial intelligence in robotic surgery technical skills assessment: a systematic review. Br J Surg.

[27] Kankanamge D, Wijeweera C, Ong Z, Preda T, Carney T, Wilson M, Preda V (2025). Artificial intelligence based assessment of minimally invasive surgical skills using standardised objective metrics - a narrative review. Am J Surg.

[28] Gao Y, Kruger U, Intes X, Schwaitzberg S, De S (2020). A machine learning approach to predict surgical learning curves. Surgery.

[29] Moglia A, Morelli L, D'Ischia R (2022). Ensemble deep learning for the prediction of proficiency at a virtual simulator for robot-assisted surgery. Surg Endosc.

[30] McCulloch RA, Howgate D, Gibbs VN, Palmer A, Taylor AH, Kendrick B (2021). Assessing the performance and learning curve of orthopaedic surgical trainees in primary total hip arthroplasty. Ann R Coll Surg Engl.

[31] Ledwos N, Mirchi N, Yilmaz R (2022). Assessment of learning curves on a simulated neurosurgical task using metrics selected by artificial intelligence. J Neurosurg.

[32] Gupta A, Kocielnik R, Wang J (2024). Multi-modal self-supervised learning for surgical feedback effectiveness assessment. ArXiv.

[33] Quick JA, Kudav V, Doty J, Crane M, Bukoski AD, Bennett BJ, Barnes SL (2017). Surgical resident technical skill self-evaluation: increased precision with training progression. J Surg Res.

[34] Walsh R, Kearns EC, Moynihan A (2023). Ethical perspectives on surgical video recording for patients, surgeons and society: systematic review. BJS Open.

[35] Prigoff JG, Sherwin M, Divino CM (2016). Ethical recommendations for video recording in the operating room. Ann Surg.

[36] Satapathy P, Hermis AH, Rustagi S, Pradhan KB, Padhi BK, Sah R (2023). Artificial intelligence in surgical education and training: opportunities, challenges, and ethical considerations - correspondence. Int J Surg.

[37] Saldanha OL, Pfeiffer K, Bodenstedt S (2025). Decentralized, privacy-preserving surgical video analysis with Swarm learning. medRxiv.

[38] Tran MT, Ahmad M, Patel K, Argyriou O, Davies A, Shalhoub J (2024). Comparing virtual reality and simulation to teach the assessment and management of acute surgical scenarios: a pilot study. Health Sci Rep.

[39] Sieck C, Rastetter M, Hefner J (2021). The five A's of access for TechQuity. J Health Care Poor Underserved.

[40] Antaki F, Doucet C, Milad D (2024). RetinaVR: Democratizing vitreoretinal surgery training with a portable and affordable virtual reality simulator in the metaverse. arXiv.

[41] Skinner J, Staiger D (2015). Technology diffusion and productivity growth in health care. Rev Econ Stat.

[42] Parham G, Bing EG, Cuevas A, Fisher B, Skinner J, Mwanahamuntu M, Sullivan R (2019). Creating a low-cost virtual reality surgical simulation to increase surgical oncology capacity and capability. Ecancermedicalscience.

